# Detection of Signals for Smell and Taste Disorders Associated with Z-drugs: A Disproportionality Analysis of the FAERS Database

**DOI:** 10.3290/j.ohpd.c_2791

**Published:** 2026-09-09

**Authors:** Hui Yang, Youde Liang, Haijing Wang, Qiang Xu, Zhengwei Kou, Yuzhen Zou

**Affiliations:** a Hui Yang Dental Practitioner, Department of Stomatology, The People’s Hospital of Baoan Shenzhen, Shenzhen, 518100 China; involved in the conceptualisation and study design, and wrote and reviewed the manuscript; b Youde Liang Dental Practitioner, Department of Stomatology, The People’s Hospital of Baoan Shenzhen, Shenzhen, 518100 China; involved in the data extraction and analysis and statistical methodology; c Haijing Wang Dental Practitioner, Department of Stomatology, The People’s Hospital of Baoan Shenzhen, Shenzhen, 518100 China; involved in the data extraction, analysis, and visualisation; d Qiang Xu Dental Practitioner, Department of Stomatology, The People’s Hospital of Baoan Shenzhen, Shenzhen, 518100 China; wrote the literature review and validated the analysis; e Zhengwei Kou* Oral and Maxillofacial Surgeon, Department of Oral and Maxillofacial Surgery, The People’s Hospital of Baoan Shenzhen, Shenzhen, 518100 China; involved in the supervision, conceptualisation, and study design, and wrote and reviewed the manuscript; f Yuzhen Zou* Dental Practitioner, Department of Stomatology, The People’s Hospital of Baoan Shenzhen, Shenzhen, 518100 China; involved in the supervision, conceptualisation, and study design, and wrote and reviewed the manuscript

**Keywords:** disproportionality analysis, dysgeusia, eszopiclone, Food and Drug Administration Adverse Event Reporting System, parosmia, pharmacovigilance, taste disorder, Z-drugs

## Abstract

**Objective:**

This study aimed to detect and characterise signals of disproportionate reporting for smell and taste disorders associated with Z-drugs in the Food and Drug Administration (FDA) Adverse Event Reporting System (FAERS).

**Methods:**

Reports involving Z-drugs and smell/taste disorders were identified using the Standardised MedDRA Query (SMQ) ‘Taste and smell disorders’. Disproportionality analyses were performed using the reporting odds ratio (ROR), proportional reporting ratio (PRR), empirical Bayes geometric mean (EBGM), and information component (IC). Sex-stratified, age-stratified, and time-to-onset (TTO) analyses were also conducted.

**Results:**

Among 32,853 reports involving Z-drugs, 3,480 were classified as smell- or taste-related adverse events. Eszopiclone accounted for 97.6% of these reports and demonstrated a strong disproportionality signal. Dysgeusia was the predominant adverse event, whereas parosmia and the preferred term ‘taste disorder’ generated additional positive signals. Signal strength was greatest among adults aged 18 to 45 years, and parosmia showed stronger disproportionality among women. Most events were reported within 30 days after treatment initiation.

**Conclusions:**

FAERS data indicate a strong association between eszopiclone and smell/taste disorders, particularly dysgeusia. Parosmia emerged as an additional signal warranting clinical attention. Further epidemiological and mechanistic studies are needed to clarify the underlying mechanisms and clinical significance of these findings.

Insomnia affects more than 16% of the global population and represents a substantial public health burden.^5^ It is associated with a wide range of adverse health outcomes, including cardiovascular, metabolic, and neurodegenerative diseases, leading to impaired daytime functioning, reduced work productivity, increased accident risk, and worsening of coexisting medical conditions.^10,12
^ Zolpidem, zaleplon, zopiclone, and eszopiclone, collectively known as Z-drugs, are non-benzodiazepine hypnotics that act as positive allosteric modulators of γ-aminobutyric acid type A (GABAA) receptors and are widely prescribed for the short-term treatment of insomnia.^6,9
^ Owing to their relatively short half-lives, Z-drugs have traditionally been considered safer than benzodiazepines; however, accumulating post-marketing evidence has identified a range of adverse effects that warrant continued pharmacovigilance.^9,14,22
^


Taste and smell disorders are clinically relevant adverse events that may negatively affect appetite, nutritional status, quality of life, and treatment adherence. Chemosensory dysfunction may additionally influence dietary behaviour and oral health-related well-being.^11,19
^ Although dysgeusia has been reported in clinical trials, case reports, and product labeling for certain Z-drugs,^8^ particularly eszopiclone, the broader spectrum of chemosensory adverse events, including anosmia, hyposmia, parosmia, ageusia, hypogeusia, and taste disorder, remains insufficiently characterised. Furthermore, little is known about their reporting patterns, demographic characteristics, or onset profiles, or differences among individual Z-drugs in real-world clinical practice.

Spontaneous reporting systems such as the US Food and Drug Administration (FDA) Adverse Event Reporting System (FAERS) play a crucial role in the detection of rare, delayed, or previously under-recognised adverse drug reactions across large and heterogeneous populations.^22^ To date, no comprehensive pharmacovigilance study has systematically evaluated smell- and taste-related adverse events associated with all marketed Z-drugs using FAERS data. Such analyses may help characterise potential safety signals, identify vulnerable populations, and provide evidence for future pharmacoepidemiological investigations.^13^


Therefore, this study aimed to evaluate reporting signals for smell and taste disorders associated with individual Z-drugs in the FAERS database using disproportionality analyses. The authors further examined demographic characteristics, seriousness outcomes, and time-to-onset patterns analyses to assess the robustness of the observed associations.

## Methods

### Study Design and Data Source

A retrospective case–non-case disproportionality analysis was conducted using publicly available reports from the FAERS. The DEMO, DRUG, REAC, OUTC, THER, and INDI tables were used. The FAERS extraction window was 2004Q1 to 2025Q2 and MedDRA version 25.0 was used (developed by the International Council for Harmonisation of Technical Requirements for Pharmaceuticals for Human Use).

### Data Extraction and Processing

#### Case identification, linkage, and deduplication

Following FDA recommendations, duplicate records in FAERS were identified and removed using CASEID-based deduplication. For cases with multiple reports, only the most recent record (based on FDA_DT, and PRIMARYID when necessary) was retained. PRIMARYID was used as the unique identifier for individual FAERS records in subsequent analyses. All database tables (DEMO, DRUG, REAC, OUTC, THER, and INDI) were linked using PRIMARYID.

#### Definition of the exposure: Z-drugs

Exposure comprised reports listing one or more of the following active ingredients: zolpidem, zopiclone, eszopiclone, and zaleplon. Generic names, brand names, and spelling variants were standardised using a curated drug dictionary. Drug identification was based on active ingredient names as recorded in FAERS.

In the primary analysis, only reports in which a Z-drug was coded as the primary suspect (PS; ROLE_COD = ‘PS’) were included to improve specificity of drug-event associations.

#### Definition of outcomes: smell and taste disorders

Primary outcomes were defined using the Standardised MedDRA Query (SMQ) ‘Taste and smell disorders’ (SMQ ID 20000046). A report was classified as a case if any preferred term (PT) in the REAC table matched the SMQ list; multiple qualifying PTs within one report were counted once. Illustrative PTs include anosmia, hyposmia, parosmia, phantosmia, ageusia, hypogeusia, and dysgeusia; the exact PT list mapped to the SMQ is provided in Supplementary Table 1. In the MedDRA terminology, the PT ‘dysgeusia’ broadly encompasses specific patient-reported lower-level terms (LLTs) such as ‘bitter taste’ and ‘metallic taste’.

**Table 1 Table1:** Two-by-two contingency table for disproportionality analysis

Item	Taste and smell disorder reported	Other adverse events reported	Total
Z-drugs	a	b	a + b
Other drugs	c	d	c + d
Total	a + c	b + d	a + b + c + d


#### Disproportionality analysis

Case–non-case two‑by‑two contingency tables were constructed for each drug-event pair using the standard approach applied to all FAERS reports as the comparator. The contingency table entries for each Z‑drug and the SMQ outcome are shown in Table 1.

Data mining employed disproportionality analyses including the reporting odds ratio (ROR), proportional reporting ratio (PRR), Bayesian confidence propagation neural network (BCPNN), and multi‑item gamma‑Poisson shrinker (MGPS).^22^ The equations and detection thresholds for each method are shown in Supplementary Table 2. Adverse‑event signals were considered present only when all four methods detected an association and a statistically significant relationship between the suspect drug and the event was observed, suggesting a potential link. Subgroup analyses were conducted by stratifying reports by sex and age group (< 18, 18 to 45, > 45 years) to evaluate potential differences in disproportionality across population segments. Within each subgroup, all four disproportionality methods (ROR, PRR, BCPNN, and MGPS) were rerun and their signal detection concordance and relative strengths were compared.

**Table 2 Table2:** Clinical characteristics of reports of Z-drug–associated taste and smell disorders

Variable		Eszopiclone		Zolpidem		Zaleplon		Zopiclone		All	
		target_PT (n = 3,398)	Overall (N = 10,839)	target_PT (n = 77)	Overall (N = 21,401)	target_PT (n = 4)	Overall (N = 518)	target_PT (n = 1)	Overall (N = 95)	target_PT (n = 3,480)	Overall (N = 32,853)
Sex	Female	2,242(66.0%)	6,577(60.7%)	49(63.6%)	11,262(52.6%)	2(50.0%)	202(39.0%)	1(100.0%)	45(47.4%)	2,294(65.9%)	18,086(55.1%)
	Male	930(27.4%)	3,476(32.1%)	19(24.7%)	7,433(34.7%)	0(0.0%)	117(22.6%)	0(0.0%)	35(36.8%)	949(27.3%)	11,061(33.7%)
	Missing	226(6.7%)	786(7.3%)	9(11.7%)	2,706(12.6%)	2(50.0%)	199(38.4%)	0(0.0%)	15(15.8%)	237(6.8%)	3,706(11.3%)
Age,y	< 18	8(0.2%)	73(0.7%)	0(0.0%)	382(1.8%)	0(0.0%)	2(0.4%)	1(100.0%)	7(7.4%)	9(0.3%)	464(1.4%)
	[18 ~ 65)	1,818(53.5%)	4,842(44.7%)	29(37.7%)	8,889(41.5%)	1(25.0%)	138(26.6%)	0(0.0%)	15(15.8%)	1,848(53.1%)	13,909(42.3%)
	[65 ~ 85]	535(15.7%)	2,548(23.5%)	16(20.8%)	3,576(16.7%)	0(0.0%)	54(10.4%)	0(0.0%)	3(3.2%)	551(15.8%)	6,193(18.9%)
	> 85	17(0.5%)	281(2.6%)	1(1.3%)	538(2.5%)	0(0.0%)	4(0.8%)	0(0.0%)	40(42.1%)	18(0.5%)	826(2.5%)
	Missing	1,020(30.0%)	3,095(28.6%)	31(40.3%)	8,016(37.5%)	3(75.0%)	320(61.7%)	0(0.0%)	30(31.6%)	1,054(30.3%)	11,461(34.9%)
Outcome	Congenitalanomaly	0 (0.0%)	1(0.0%)	0(0.0%)	69(0.3%)	0(0.0%)	5(1.0%)	0(0.0%)	1(1.1%)	0(0.0%)	71(0.2%)
	Death	0(0.0%)	88(0.8%)	0(0.0%)	2,157(10.1%)	0(0.0%)	1(0.2%)	0(0.0%)	23(24.2%)	0(0.0%)	2,273(6.9%)
	Disablity	1(0.0%)	14(0.1%)	1(1.3%)	154(0.7%)	0(0.0%)	12(2.3%)	0(0%)	3(3.2%)	2(0.1%)	172(0.5%)
	Hospitalisation	0(0.0%)	134(1.2%)	5(6.5%)	3,584(16.7%)	0(0.0%)	2(0.4%)	0(0.0%)	29(30.5%)	5(0.1%)	3,759(11.4%)
	Life-threatening	1(0.0%)	29(0.3%)	1(1.3%)	906(4.2%)	4(100%)	498(96.1%)	0(0.0%)	5(5.3%)	2(0.1%)	942(2.9%)
	Other	3,396(99.9%)	10,573(97.5%)	70(90.9%)	14,531(67.9%)	0(0.0%)	5(1.0%)	1(100.0%)	34(35.8%)	3,471(99.7%)	25,636(78.0%)
Seriouscases	No	3,385(99.6%)	10,285(94.9%)	54(70.1%)	10,244(47.9%)	3(75.0%)	461(89.0%)	1(100.0%)	10(10.5%)	3,443(98.9%)	21,000(63.9%)
	Yes	13(0.4%)	554 (5.1%)	23(29.9%)	11,157(52.1%)	1(25.0%)	57(11.0%)	0(0.0%)	85(89.5%)	37(1.1%)	11,853(36.1%)
Fatalcases	No	3,398(100.0%)	10,751(99.2%)	77(100%)	19,244(89.9%)	4(100%)	513(99.0%)	1(100.0%)	72(75.8%)	3,480(100%)	30,580(93.1%)
	Yes	0(0.0%)	88(0.8%)	0(0.0%)	2,157(10.1%)	0(0.0%)	5(1.0%)	0(0.0%)	23(24.2%)	0(0.0%)	2,273(6.9%)
Reportertype	Consumer	2,716(79.9%)	8,020(74.0%)	53(68.8%)	7,650(35.7%)	3(75.0%)	365(70.5%)	1(100.0%)	28(29.5%)	2,773(79.7%)	16,063(48.9%)
	Healthprofessional	5(0.1%)	46(0.4%)	1(1.3%)	1,484(6.9%)	0(0.0%)	6(1.2%)	0(0.0%)	5(5.3%)	6(0.2%)	1,541(4.7%)
	Pharmacist	115(3.4%)	442(4.1%)	4(5.2%)	2,603(12.2%)	1(25.0%)	35(6.8%)	0(0.0%)	7(7.4%)	120(3.4%)	3,087(9.4%)
	Physician	293(8.6%)	1,235(11.4%)	4(5.2%)	4,684(21.9%)	0(0.0%)	54(10.4%)	0(0.0%)	18(18.9%)	297(8.5%)	5,991(18.2%)
	Missing	269(7.9%)	1,096(10.1%)	15(19.5%)	4,980(23.3%)	0(0.0%)	58(11.2%)	0(0.0%)	37(38.9%)	284(8.2%)	6,171(18.8%)


#### Time-to-onset (TTO) analysis

When both START_DT (drug start date) and EVENT_DT (event onset date) were available and plausible (EVENT_DT ≥ START_DT and dates passing logical checks), time‑to‑onset (TTO) in days was calculated as TTO = EVENT_DT-START_DT. Reports with missing or implausible dates were excluded from TTO summaries but retained for disproportionality analyses.

#### Missing data handling

Missingness in covariates was summarised; no statistical imputation was performed. ‘Unknown’ categories were retained for transparency and used in analyses where relevant.

## Results

### Clinical Characteristics of Reports of Taste and Smell Disorders Associated with Z-drugs

A total of 32,853 reports involving Z-drugs were identified between 1 January 2004 and 30 June 2025, of which 3,480 (10.6%) met the predefined criteria for smell and taste disorders based on the SMQ ‘taste and smell disorders’. Nearly all cases were associated with eszopiclone (3,398 reports, 97.6%), followed by zolpidem (77 reports, 2.2%), zaleplon (4 reports, 0.1%), and zopiclone (1 report, < 0.1%).

Female patients accounted for 66.0% of reports, compared with 27.4% for male patients, while 6.7% had missing sex information. Among reports with available age data, adults aged 18 to 64 years constituted the largest age group. Most reports were classified as non-serious (99.6%), whereas only 0.4% were considered serious. No fatal cases were identified among reports of smell and taste disorders. Consumer reports predominated (79.9%), followed by physicians (8.6%), pharmacists (3.4%), and other health care professionals (0.1%) (Table 2 and Fig 1).

**Fig 1 Fig1:**
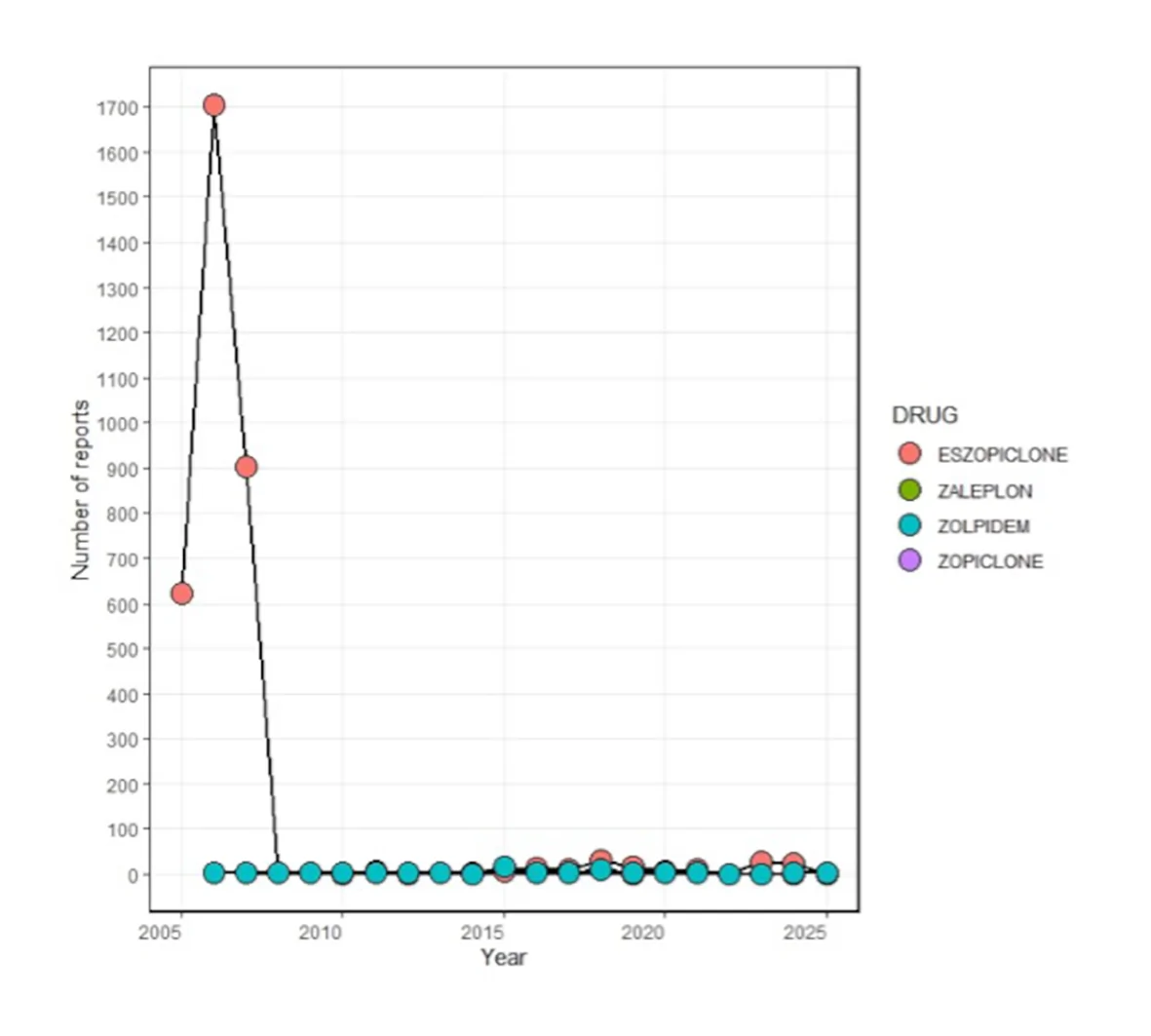
Temporal distribution of reports of Z‑drug–associated taste and smell disorders.

### Disproportionality Analysis of Taste and Smell Disorders Associated with Z-drugs

Among the four evaluated Z-drugs, only eszopiclone demonstrated a robust disproportionality signal for smell and taste disorders. A total of 3398 reports were identified, yielding a ROR of 81.42 (95% confidence interval [CI] 78.47 to 84.48), a PRR of 68.76, an empirical Bayes geometric mean (EBGM) of 66.84 (EB05 = 64.42), and an information component (IC) of 6.06 (IC025 = 5.98), indicating a very strong reporting signal. In contrast, zolpidem showed no evidence of disproportional reporting (ROR 0.52, 95% CI 0.42 to 0.65; IC025 = −1.26) (Table 3). Similarly, no statistically meaningful signals were detected for zaleplon or zopiclone, although interpretation was limited by the small number of reports associated with these agents. Overall, the observed signal for smell and taste disorders among Z-drugs was driven almost entirely by eszopiclone, which accounted for 97.6% of all identified cases (Table 3).

**Table 3 Table3:** Disproportionality analysis of taste and smell disorders with Z-drugs

Drug name	Case reports (n)	ROR (95% CI)	PRR (χ^2^)	EBGM (EBGM05)	IC (IC025)
Eszopiclone	3,398*	81.42 (78.47–84.48)	68.76 ( 222,870.55)	66.84 (64.42)	6.06 (5.98)
Zolpidem	77	0.52 (0.42–0.65)	0.52 (35.26)	0.52 (0.42)	−0.95 (−1.26)
Zaleplon	4	1.60 (0.6–4.27)	1.6 ( 0.89)	1.6 (0.60)	0.67 (−0.78)
Zopiclone	1	1.30 (0.1–9.28)	1.3 (0.07)	1.3 (0.18)	0.38 (1.87)
*Statistically significant.					

### Eszopiclone-specific PT-level Disproportionality Analysis

Because eszopiclone accounted for 97.6% of all identified smell- and taste-related reports and was the only Z-drug that demonstrated a robust SMQ-level signal, subsequent PT-level analyses were restricted to eszopiclone (Table 4). Among the evaluated PTs, dysgeusia was by far the most frequently reported event, accounting for 3,377 reports. It showed an exceptionally strong disproportionality signal (ROR 157.03, 95% CI 151.55–162.71; IC025 = 6.97). Parosmia also demonstrated a significant reporting signal (ROR 8.56, 95% CI 5.78 to 12.67; IC025 = 3.09), whereas the PT ‘taste disorder’ exhibited a weaker but positive signal (ROR 2.00, 95% CI 1.18 to 3.38; IC025 = 1.00). In contrast, anosmia, ageusia, and olfactory hallucination did not meet the predefined signal detection criteria. Overall, the PT-level analysis indicated that the observed chemosensory safety signal associated with eszopiclone was overwhelmingly driven by dysgeusia, while parosmia and nonspecific taste disorder represented less frequent but statistically significant signals (Fig 2a).

**Table 4 Table4:** Eszopiclone-specific PT-level disproportionality analysis

PT	ROR (95% CI)	PRR (χ^2^)	EBGM (EBGM05)	IC (IC025)
Anosmia	0.18 (0.02–1.25)	0.18 (3.86)	0.18 (0.02)	−2.51 (−3.78)
Ageusia	1.42 (0.84 –2.4)	1.42 (1.76)	1.42 (0.84)	0.51 (−0.28)
Parosmia	8.56 (5.78–12.67)*	8.55 (166.05)*	8.52 (5.75)*	3.09 (2.16)*
Taste disorder	2 (1.18–3.38)*	2 (6.99)*	2 (1.18)	1 (0.16)*
Dysgeusia	157.03 (151.55–162.71)*	132.64 (467,936.61) *	125.61 (121.23) *	6.97 (6.87)*
Hallucination, olfactory	6.04 (0.85–43.01 )	6.04 (4.20)	6.03 (0.85)	2.59 (−1.27)
*Statistically significant.				

**Fig 2 Fig2:**
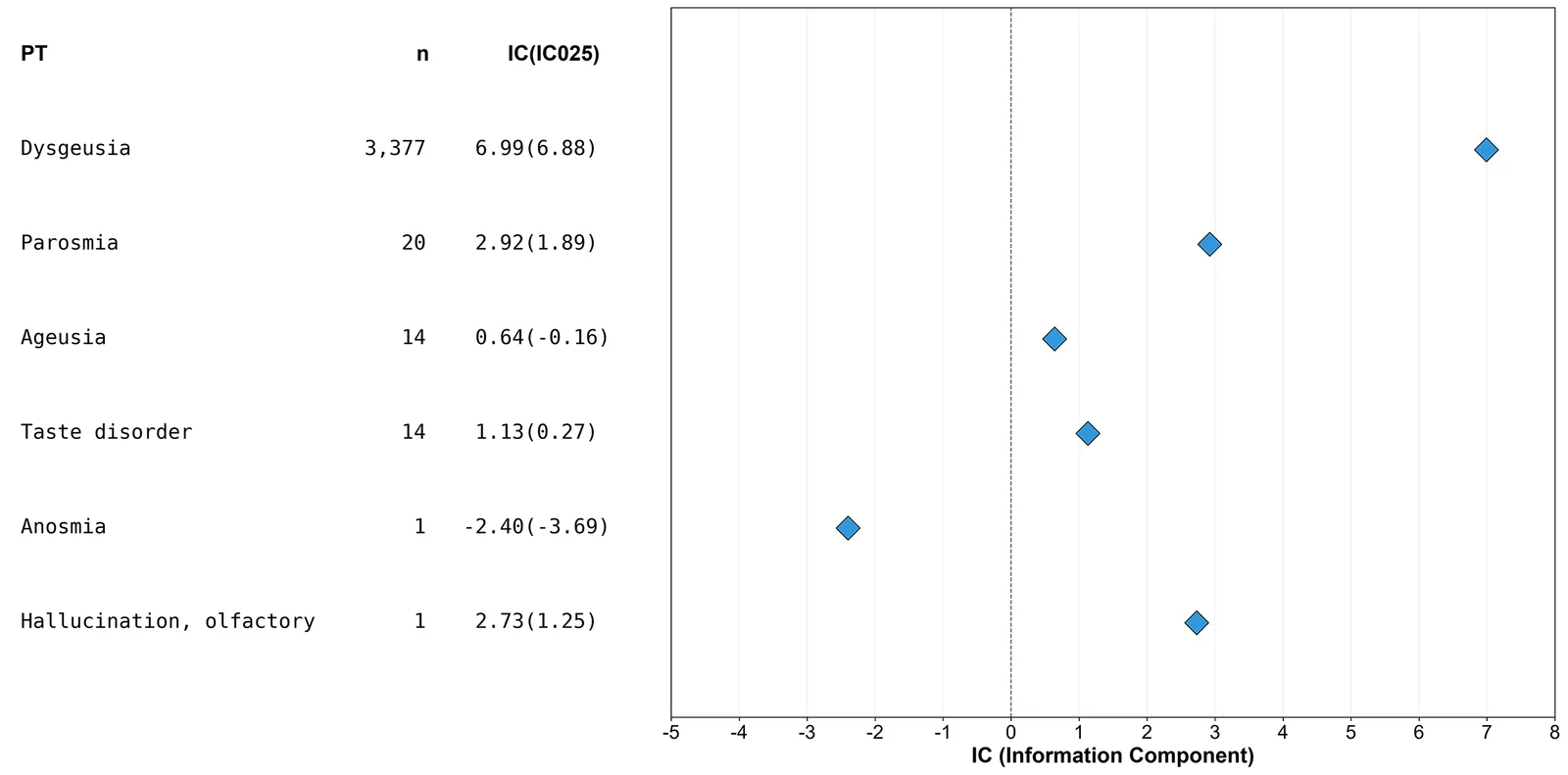

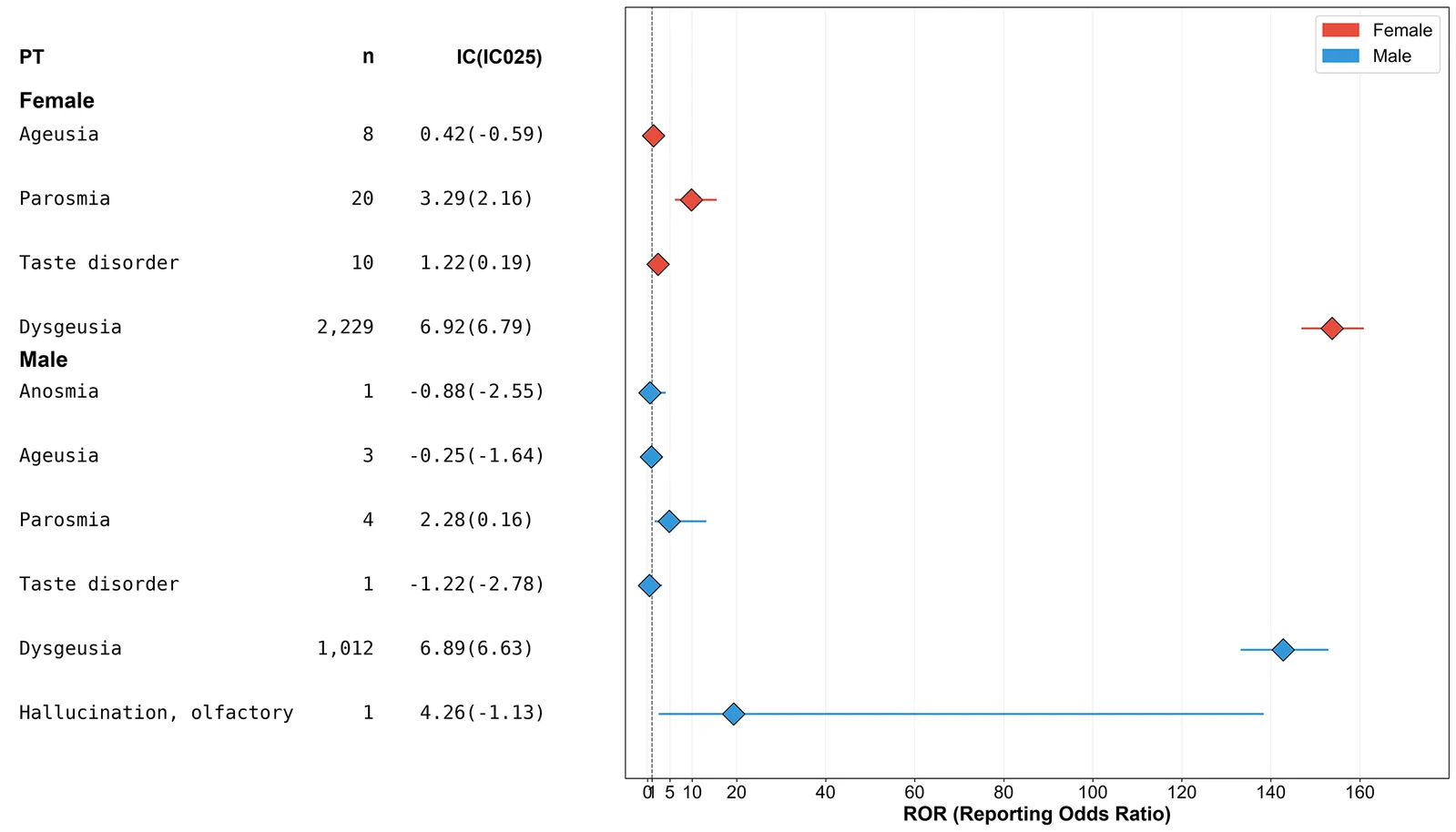

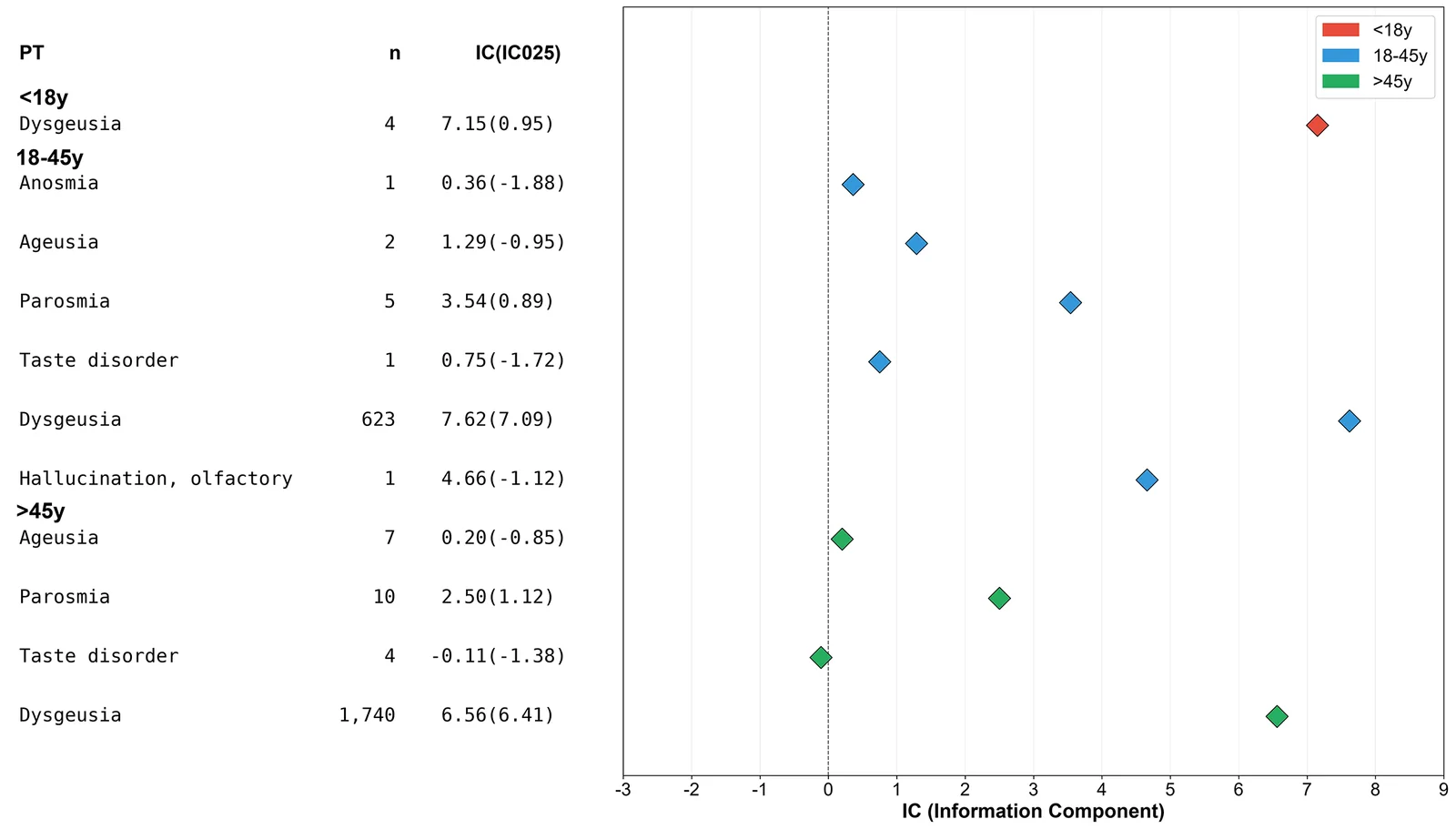
Sex-and age stratified RORs for eszopiclone-associated taste and smell disorders. RORs (a); sex-stratified RORs (b); age-stratified RORs (c). Point estimates (squares) and 95% confidence intervals (horizontal bars); x-axis on log scale; vertical dashed line at ROR = 1. Arrows indicate CIs truncated at axis limits. CI_text, 95% credibility interval.

### Sex-stratified Disproportionality Analyses of Eszopiclone 

Dysgeusia remained the predominant signal in both females and males, with strong and comparable disproportionality estimates across sexes (female ROR 153.74, 95% CI 147.12 to 160.66; male ROR 142.77, 95% CI 133.45 to 152.74).

Parosmia demonstrated positive disproportionality signals in both sexes, although the signal was stronger among female participants (female ROR 9.85, 95% CI 6.35 to 15.29; male ROR 4.86, 95% CI 1.82 to 12.96). In contrast, the PT ‘taste disorder’ met the predefined signal detection criteria only among female participants (ROR 2.33, 95% CI 1.26 to 4.34), whereas no significant signal was observed among male participants.

Ageusia, anosmia, and olfactory hallucination were infrequently reported and did not demonstrate robust disproportionality signals. Overall, dysgeusia represented the dominant adverse event regardless of sex, while parosmia showed stronger disproportionality among female participants and the PT ‘taste disorder’ generated a detectable signal only in female reports (Fig 2b).

### Age-stratified Disproportionality Analyses of Eszopiclone

Dysgeusia remained the predominant signal across all age groups, although signal strength varied substantially by age. The 18- to 45-year age group exhibited the strongest disproportionality signal for dysgeusia (ROR 266.51, 95% CI 243.38 to 291.85), based on 623 reports, whereas the corresponding signal was lower in individuals older than 45 years (ROR 114.35, 95% CI 108.60 to 120.41). Although the < 18-year age group also showed a positive signal, only four reports were identified, and the resulting estimates should therefore be interpreted with caution.

Parosmia demonstrated a similar age-related pattern, with a stronger signal observed among individuals aged 18 to 45 years (ROR 11.70, 95% CI 4.86 to 28.19) than among those older than 45 years (ROR 5.69, 95% CI 3.06 to 10.60). In contrast, ageusia and the PT ‘taste disorder’ did not meet the predefined signal detection criteria in any age stratum.

Overall, disproportionality signals for eszopiclone-associated dysgeusia and parosmia were strongest among younger adults, whereas weaker signals were observed in older individuals (Fig 2c).

### TTO Analyses for Eszopiclone-associated Taste and Smell Disorders

Among 979 eszopiclone–dysgeusia reports considered for TTO assessment, valid onset intervals were available for 442 reports. A total of 537 reports were excluded because of missing, incomplete, or illogical date information. The median TTO was 1 days (interquartile range [IQR] 1 to 17.5 days). The largest proportion of reports occurred within the first 30 days after treatment initiation (77.8%), followed by the 181- to 360-day interval (8.1%). Although reporting was concentrated during the early treatment period, smell- and taste-related adverse events were observed throughout the course of treatment (Fig 3).

**Fig 3 Fig3:**
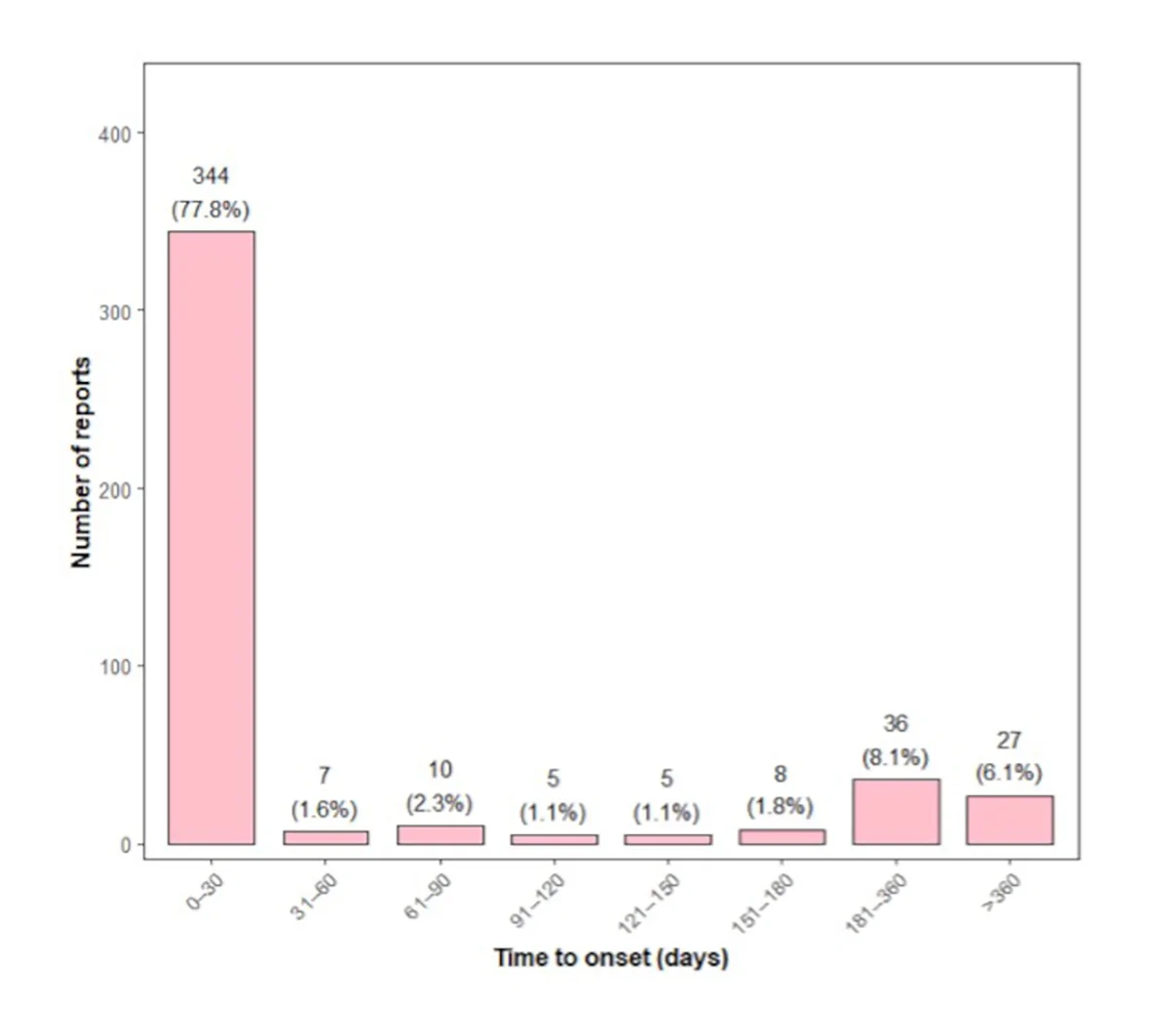
TTO analyses for taste and smell disorders associated with eszopiclone.

## Discussion

In this large pharmacovigilance study based on FAERS data, eszopiclone was the only Z-drug associated with a robust and consistent disproportionality signal for smell and taste disorders. Dysgeusia accounted for nearly all reported chemosensory adverse events and exhibited extremely strong disproportionality across multiple signal detection methods. Additional signals were identified for parosmia and non-specific taste disorder. Furthermore, signal strength appeared greater among younger adults, and most reports occurred within the first month following treatment initiation.

The predominance of eszopiclone was not unexpected. Dysgeusia is a recognised adverse reaction described in the prescribing information for eszopiclone and has been documented in clinical trials and post-marketing reports.^8^ Experimental evidence suggests that eszopiclone-associated dysgeusia may be influenced by sex, time since dosing, and blood and saliva concentrations of the drug, with women reporting greater frequency and intensity and a positive correlation between symptom intensity and eszopiclone levels.^21^ In this pharmacovigilance study, younger adults demonstrated stronger disproportionality signals than older individuals. The strongest signals were observed in the 18- to 45-year age group. From a physiological perspective, older adults often experience natural taste bud atrophy and declining sensory function, which might obscure drug-induced changes.^16^ In addition, the temporal concentration of eszopiclone-related reports during 2005 to 2007 should be interpreted in the context of the Weber effect,^3^ a well-recognised pharmacovigilance phenomenon in which spontaneous adverse event reporting often peaks within the first few years after market approval and declines thereafter. Because eszopiclone was approved by the FDA in late 2004, the early post-marketing peak observed in our analysis may reflect heightened clinical awareness, stimulated reporting, and early market uptake rather than a temporal change in biological susceptibility.

The mechanism of underlying eszopiclone-associated smell and taste disorders remains incompletely understood. Eszopiclone or its metabolites may act on oral or nasal mucosa, alter saliva secretion, or affect sensory epithelium function, thereby modifying taste and smell perception. Saliva plays a critical role in tastant diffusion, receptor stimulation, and mucosal protection; consequently, drug-induced xerostomia may contribute to taste disturbances.^17^ Approximately 45% of medications known to cause taste disorders have also been associated with dry mouth,^20,22
^ and xerostomia has been reported with eszopiclone use.^7,15
^ In addition, eszopiclone is known to produce a characteristic bitter or metallic taste, which may be related to drug secretion into saliva shortly after administration. Direct exposure of taste buds and oral mucosal receptors to salivary eszopiclone may therefore contribute to local taste disturbances, either independently of or in combination with central nervous system mechanisms.^18^ Furthermore, an association between non-benzodiazepine hypnotics and oral soft tissue infections has been reported^22^ and could potentially contribute to sensory epithelial damage in some patients.^4^ These hypotheses warrant further pharmacological, physiological, and histological investigation.

Beyond dysgeusia, a positive disproportionality signal was also observed for parosmia. Although the number of reports was considerably smaller, the signal remained detectable across signal detection algorithms. This finding expands the spectrum of potential chemosensory adverse effects associated with eszopiclone and suggests that drug-related sensory disturbances may involve not only altered taste perception but also abnormalities in odor perception.

### Clinical Implications

Clinically, heightened multidisciplinary vigilance for taste and smell alterations is recommended after initiation of Z drugs, particularly eszopiclone. In addition to prescribers of sedative-hypnotics, dental and oral health professionals should be aware that unexplained taste disturbances may be temporally related to medication exposure. Reviewing medication history, particularly recent or current Z-drug use, may help distinguish potential drug-related taste complaints from primary dental or oral pathology and may reduce unnecessary dental interventions.

### Strengths and Limitations 

This study leveraged a large national spontaneous reporting database and applied multiple complementary disproportionality methods, together with TTO description, PT level, and subgroup (sex, age) stratified analyses to improve signal robustness and explore heterogeneity. However, several limitations should be acknowledged. First, FAERS is a spontaneous reporting system and is subject to underreporting, duplicate reporting, stimulated reporting, missing information, and reporting bias. Disproportionality analysis is intended for signal detection and cannot establish causality, clinical association, or absolute risk. Therefore, the signals of disproportionate reporting identified in this study should be interpreted as potential safety signals rather than evidence that Z-drugs cause smell or taste disorders. Second, FAERS lacks a reliable denominator representing the total number of exposed patients for each drug; consequently, true incidence rates and comparative risks cannot be calculated. Third, TTO analyses may be affected by missing, incomplete, or inaccurate date information, and reports with unavailable or illogical dates were excluded. Finally, information on relevant clinical factors such as oral health status, salivary flow, smoking, COVID-19 infection, neurological disease, psychiatric comorbidity, and concomitant xerogenic medications was incomplete or unavailable, limiting the present authors’ ability to evaluate confounding factors.

## Conclusion

In summary, FAERS data identified a strong disproportionality signal between eszopiclone and smell/taste disorders, particularly dysgeusia. Parosmia and the PT ‘taste disorder’ also generated positive signals, thereby expanding the spectrum of potential chemosensory adverse events associated with eszopiclone. Most events were reported shortly after treatment initiation. Because disproportionality analyses are hypothesis-generating rather than confirmatory, further epidemiological studies with defined exposure denominators and mechanistic investigations are needed to verify these associations, clarify causality, and identify susceptible patient populations.

## References

### APPENDIX

**Table A1 TableA1:** PTs (MedDRA version 25.0) of taste and smell disorders

PT name	PT code	Number of records
Dysgeusia	10013911	3,440
Ageusia	10001480	22
Taste disorder	10082490	16
Anosmia	10002653	3
Parosmia	10034018	27
Hypogeusia	10020989	0
Hyposmia	10050515	0
Hallucination, olfactory	10019072	2
Olfactory nerve disorder	10056388	1
Hypergeusia	10069147	0
Hallucination, gustatory	10019071	0
Gustometry abnormal	10064480	0
Olfactory test abnormal	10062927	0
Olfactory dysfunction	10086567	0




## References

[ref1] Arora A, Jalali RK, Vohora D (2017). Relevance of the Weber effect in contemporary pharmacovigilance of oncology drugs. Ther Clin Risk Manag.

[ref2] Atukorallaya DS,  Ratnayake RK (2021). Oral mucosa, saliva, and COVID-19 infection in oral health care. Front Med.

[ref3] Benjafield AV, Sert Kuniyoshi FH, Malhotra A (2025). Estimation of the global prevalence and burden of insomnia: a systematic literature review-based analysis. Sleep Med Rev.

[ref4] Brandt J, Leong C (2017). 2017. Benzodiazepines and Z-drugs: An updated review of major adverse outcomes reported on in epidemiologic research. Drugs R D.

[ref5] Doty RL, Treem J, Tourbier I, Mirza N (2009). A double-blind study of the influences of eszopiclone on dysgeusia and taste function. Pharmacol Biochem Behav.

[ref6] Gunja N (2013). The clinical and forensic toxicology of Z-drugs. J Med Toxicol.

[ref7] Ishak WW, Bagot K, Thomas S (2012). Quality of life in patients suffering from insomnia. Innov Clin Neurosci.

[ref8] Kan Y, Nagai J, Uesawa Y (2021). Evaluation of antibiotic-induced taste and smell disorders using the FDA adverse event reporting system database. Sci Rep.

[ref9] Kessler RC, Berglund PA, Coulouvrat C (2011). Insomnia and the performance of US workers: Results from the America insomnia survey. Sleep.

[ref10] Kirkwood C, Breden E (2010). Management of insomnia in elderly patients using eszopiclone. Nat Sci Sleep.

[ref11] Kyotani Y, Nakahira K, Yoshizumi M (2025). Disproportionality analysis of taste disorders using the FDA adverse event reporting system and Japanese adverse drug event report databases. Front Pharmacol.

[ref12] Leufkens TRM, Vermeeren A (2014). Zopiclone’s residual effects on actual driving performance in a standardized test: A pooled analysis of age and sex effects in 4 placebo-controlled studies. Clin Ther.

[ref13] Liang L, Huang Y, Xu R, Wei Y, Xiao L, Wang G (2019). Eszopiclone for the treatment of primary insomnia: A systematic review and meta-analysis of double-blind, randomized, placebo-controlled trials. Sleep Med.

[ref14] Liu F, Yin J, Wang J, Xu X (2022). Food for the elderly based on sensory perception: A review. Curr Res Food Sci.

[ref15] Matsuo R (2000). Role of saliva in the maintenance of taste sensitivity. Crit Rev Oral Biol Med.

[ref16] Pinto LR Jr, Azeredo Bittencourt LR, Treptow EC, Braga LR, Tufik S (2016). 2016. Eszopiclone versus zopiclone in the treatment of insomnia. Clinics.

[ref17] Rademacher WMH, Aziz Y, Hielema A (2020). Oral adverse effects of drugs: Taste disorders. Oral Dis.

[ref18] Shu Y, Ding Y, Liu Y, Wu P, He X, Zhang Q (2022). Post-marketing safety concerns with Secukinumab: A disproportionality analysis of the FDA adverse event reporting system. Front Pharmacol.

[ref19] Silva NSV, da Silva LA, Jaluul O, Jacob-Filho W, de Siqueira SRDT (2017). Oral infections, comorbidities and sensory evidences in elderly: Cross-sectional study. Arch Gerontol Geriatr.

[ref20] Treves N, Perlman A, Kolenberg Geron L, Asaly A, Matok I (2018). Z-drugs and risk for falls and fractures in older adults—A systematic review and meta-analysis. Age Ageing.

[ref21] Wolff A, Joshi RK, Jörgen Ekström J (2017). A guide to medications inducing salivary gland dysfunction, xerostomia, and subjective sialorrhea: A systematic review sponsored by the World Workshop on Oral Medicine VI. Drugs R D.

